# First Look at Chemopreventive Properties of *Chlorella pyrenoidosa* Water Extract in Human Endometrial Adenocarcinoma Cells—Preliminary In Vitro Study

**DOI:** 10.3390/ijms26189142

**Published:** 2025-09-19

**Authors:** Weronika Rzeska, Michał Chojnacki, Aneta Adamiak-Godlewska, Andrzej Semczuk, Marta Kinga Lemieszek

**Affiliations:** 1 Doctoral School of Medical Sciences, Lublin Medical University, 20-093 Lublin, Poland; 2 Department of Medical Biology, Institute of Rural Health, Jaczewskiego 2, 20-090 Lublin, Poland; 3 IInd Chair and Department of Gynaecology, Lublin Medical University, 20-090 Lublin, Poland

**Keywords:** *Chlorella pyrenoidosa*, functional food, chemoprevention, Chlorella Growth Factor, endometrial cancer

## Abstract

Chlorella species are classified as functional food, with great anticancer effects. Despite the huge popularity of Chlorella-based products, there is a lack of evidence showing their usefulness in the prevention and treatment of endometrial cancer. The study presented here aimed to enrich knowledge resources in this area. The chemopreventive effect of water extracts of *Chlorella pyrenoidosa* was investigated in human endometrial adenocarcinoma HEC-1-B, KLE and EDC cells using MTT, BrdU, LDH, Wound assays, Cell Death Detection ELISA and nuclear double staining. *C. pyrenoidosa* extract inhibited the metabolic activity, DNA synthesis and migratory capacity of endometrial cancer cells. Moreover, the extract eliminated cancer cells, causing damage to their cell membranes and inducing apoptosis. The cells most resistant to chlorella extract were EDC cells (low grade), while the best response to the treatment was noted in KLE cells (high grade). The performed study revealed the chemopreventive properties of *C. pyrenoidosa* extract based on inhibition of endometrial cancer cell viability, proliferation and migratory capacity, as well as induction of cytotoxicity and apoptosis. Collected data suggested enhancement of extract chemopreventive properties with increasing advancement and malignancy of cancer cells. Obtained results encourage future clinical research and detailed chemical evaluation to specify the extract’s phytochemical composition.

## 1. Introduction

Endometrial cancer is the most common gynecologic cancer in developed countries, accounting for approximately 7% of all female cancers [1]. The global incidence of endometrial cancer reveals an increasing trend, with nearly 417,000 new cases and 97,000 fatalities reported in 2020. The age-standardized incidence rate was 8.7 per 100,000 women, and the mortality rate was 1.8 per 100,000. The incidence has been rising steadily, with a 132% increase over the past 30 years, largely due to aging populations and increasing rates of obesity and diabetes [2,3]. The standard treatment for endometrial cancer is surgery, typically involving total hysterectomy and bilateral salpingo-oophorectomy. Adjuvant therapies, such as radiation, chemotherapy and hormonal therapy, are considered based on the stage and risk of recurrence [4]. Nevertheless, the therapeutic effects are still unsatisfactory, mostly because of cancer molecular heterogeneity, tendency to metastasis and recurrence as well as drug resistance [4,5,6]. In the face of disappointment with the effectiveness of conventional therapies and the numerous side effects that significantly reduce the comfort and quality of life of oncology patients, chemoprevention based on substances of natural origin is becoming increasingly popular. Recently, *Chlorella* sp. in particular has attracted special attention in this field of research. So far, several studies have revealed direct anticancer effects of various *Chlorella* species [7,8,9,10,11,12,13,14,15,16,17,18,19,20,21,22,23,24,25,26,27]. They have also proven the antioxidant [28,29,30,31,32,33] and immunoenhancement properties of chlorella products [29,34,35,36,37,38,39,40], which also improved patient cancer fighting abilities. Next to the above-mentioned anticancer properties of products and compounds obtained from different *Chlorella* species, their use in cancer chemoprevention is also supported by their valuable nutrient compositions. They are rich in proteins, essential amino acids, polysaccharides, pigments, unsaturated fatty acids, vitamins, minerals and other nutrients with comprehensive and balanced nutritional values and health benefits [29,41,42,43,44,45]. Taking the above into account, *Chlorella* species are classified as functional foods with great anticancer effects, and thus their consumption can be a great therapeutic strategy, especially in the case of diet-dependent cancers, among which endometrial cancer is also included.

Unfortunately, there is a lack of evidence showing the usefulness of chlorella products in the prevention and treatment of endometrial cancer. The only data dedicated to uterus cancer, which shows an antiproliferative effect of chlorella compounds, comes from the human cervical adenocarcinoma cell line HeLa [8,9,22]. Using the PrestoBlue reagent, Sawasdee et al. have shown that the ethanol extract of *Chlorella* sp. AARL G049 significantly reduced the viability of HeLa cells and the observed effect intensified with time of exposure. The half-maximal cytotoxicity concentrations (CC50) calculated based on the results obtained after 24 and 48 h of cell treatment with the tested extract were 1.13 mg/mL and 0.99 mg/mL, respectively [8]. Studies conducted by Kyadari et al. have shown that organic extract (dichloromethane/methanol; 2:1) of *C. pyrenoidosa* inhibited the viability of HeLa cells, and the half-maximal inhibitory concentration (IC50) of the extract, calculated based on MTT results after 24 h of treatment, was 570 μg/mL [22]. Hamouda et al. [9] using the MTT assay, also proved the anticancer effect of methanol extracts of *C. vulgaris* against HeLa cells. Their study revealed that extracts in concentrations of 100 µg/mL and 800 µg/mL reduced uterus cancer cell viability by 80.1% and 98.1%, respectively.

As mentioned above, there are a few scientific reports focused on the influence of chlorella-based products on the development of uterine cancer, but there is a lack of evidence strictly dedicated to endometrial cancer. Thus, the study presented here aimed to fill this knowledge gap. The study was conducted in human endometrial adenocarcinoma cell lines HEC-1-B and KLE, as well as human endometrial adenocarcinoma-derived EDC cells. Water extract of *C. pyrenoidosa* was assessed in terms of its cytotoxicity, antiproliferative properties, cell death induction and influence on cancer cell migratory capacity. The presented study is a preliminary evaluation of the chemopreventive potential of *C. pyrenoidosa* compounds in the context of their possible use in therapy for endometrial cancer. Although the study was conducted only on cell lines, the lack of data describing the anticancer properties of chlorella in the context of endometrial cancer makes it unique.

## 2. Results

### 2.1. Main Components of Water Extract of Chlorella pyrenoidosa

Using spectrophotometric techniques, the total contents of proteins, sugar and nucleic acids were determined. The performed studies revealed that chlorella extract consisted mainly of sugars (42.32%), proteins (26.86%) and nucleic acids (24.43%) (Table 1).

### 2.2. Antiproliferative Effect of Water Extract of Chlorella pyrenoidosa

**Figure 1:**
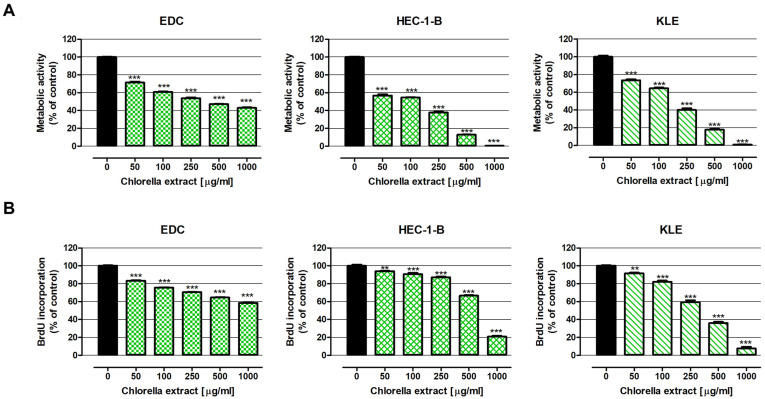
Antiproliferative Effect of C. pyrenoidosa Extract in Human Endometrial Adenocarcinoma-derived EDC Cells and Human Endometrial Adenocarcinoma Cell Lines HEC-1-B and KLE. Cells were exposed to a culture medium alone (control), or chlorella extract at concentrations of 50, 100, 250, 500 and 1000 μg/mL. The cell metabolic activity was determined after 96 h of treatment using the MTT assay (**A**), while DNA synthesis was assessed after 48 h of cell incubation with the extract using the BrdU test (**B**). Results are presented as mean ± SEM of at least 4 measurements. Statistically significant differences compared to the control at *p* < 0.01 (**), *p* < 0.001 (***). One-way ANOVA test; post-hoc test: Dunnett. Numerical data are presented in Table S1.

As presented in Figure 1A, chlorella extract in the whole range of analysed concentrations significantly decreased the metabolic activity of human endometrial adenocarcinoma cell lines KLE and HEC-1-B, as well as human endometrial adenocarcinoma-derived EDC cells. Inhibition of cancer cell viability intensified in an extract dose-dependent manner. The most sensitive cells to chlorella treatment were HEC-1-B cells, whose metabolic activity in response to the extract at a concentration of 50 µg/mL decreased by 43.2%, while after treatment with 1000 µg/mL they decreased by 99.6%. Almost complete inhibition of cancer cell metabolic activity was also observed in KLE cells exposed to chlorella extract at a concentration of 1000 µg/mL. On the contrary, EDC cells were the most resistant to the investigated extract, with their viability being reduced by 57.0% at the highest tested concentration The described changes were reflected in IC50 values, which were as follows: IC50 _MTT 96h EDC_ = 388.6 µg/mL, IC50 _MTT 96h HEC-1-B_ = 95.3 µg/mL and IC50 _MTT 96h KLE_ = 150.2 µg/mL.

The antiproliferative abilities of chlorella extract were additionally examined using a more specific and sensitive BrdU assay (Figure 1B). The tests performed showed that chlorella extract in the whole range of investigated concentrations significantly decreased DNA synthesis in the investigated cell lines and the observed effect was dose dependent. The strongest inhibition of cell proliferation was noted in KLE cells, whose divisions after treatment with chlorella extract lowered from 91.6% (50 µg/mL) to 7.7% (1000 µg/mL). Less sensitive to the antiproliferative influence of chlorella compounds were HEC-1-B cells, whose proliferation after exposure to the extract at the lowest and highest investigated concentrations decreased by 6.0% and 79.1%, respectively. Once again, EDC cells were the most resistant to the tested extract, which, at a concentration of 1000 µg/mL, inhibited DNA synthesis by 41.4%. IC50 values calculated based on the results of the BrdU assay were as follows: IC50 _BrdU 48h EDC_ =2013.0 µg/mL, IC50 _BrdU 48h HEC-1-B_ = 619.7 µg/mL and IC50 _BrdU 48h KLE_ = 305.7 µg/mL.

### 2.3. Antimigratory Effect of Water Extract of Chlorella pyrenoidosa

**Figure 2:**
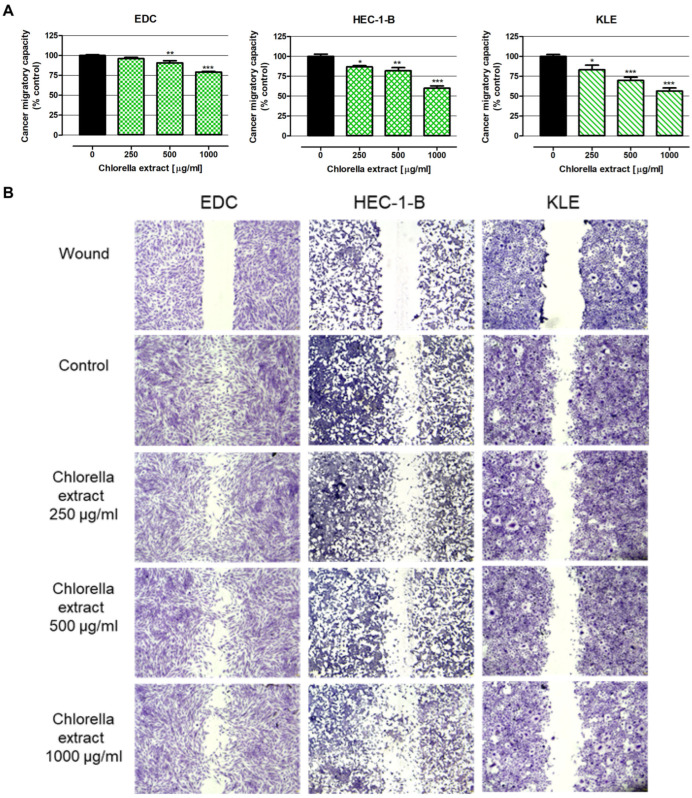
Antimigratory Effect of *C. pyrenoidosa* Extract in Human Endometrial Adenocarcinoma-derived EDC Cells and Human Endometrial Adenocarcinoma Cell Lines HEC-1-B and KLE. The monolayers of cancer cells generated in scratch wounds were incubated with culture medium alone (control) or chlorella extract in concentrations of 250, 500 and 1000 μg/mL. After 48 h of treatment, EDC, HEC-1-B and KLE cells were stained with the May–Grünwald–Giemsa method. The migratory capacity of cancer cells was determined by examination of the wound area overgrown with cells in response to the extract using Image J software version 1.53k. (**A**) Results are presented as mean ± SEM of 4 measurements. Statistically significant differences compared to the control at *p* < 0.05 (*), *p* < 0.01 (**), *p* < 0.001 (***). One-way ANOVA test; post-hoc test: Dunnett. (**B**) Micrographs show wound and cell migration in cultures treated with chlorella extract. Magnification 40×. Numerical data are presented in Table S2.

Wound assays revealed the inhibition of the migratory capacity of endometrial cancer cells in response to chlorella extract (Figure 2). The strongest response was noted in KLE cell lines, wherein the amounts of cells that colonized the scratch significantly decreased with increasing extract concentration, reaching the following values: 83.2% (250 µg/mL), 69.7% (500 µg/mL) and 56.4% (1000 µg/mL). HEC-1-B cells were also sensitive to the antimigratory influence of the tested extract, which inhibited their movement by up to 86.7% (250 µg/mL), 81.9% (500 µg/mL) and 60.0% (1000 µg/mL). Among the investigated cell lines, EDC was the most resistant to the antimigratory effect of chlorella extract. EDC migratory capacity in response to the extract at higher concentrations was 90.8% (500 µg/mL) and 79.2% (1000 µg/mL).

### 2.4. Cancer Cell Death Induction by Water Extract of Chlorella pyrenoidosa

**Figure 3:**
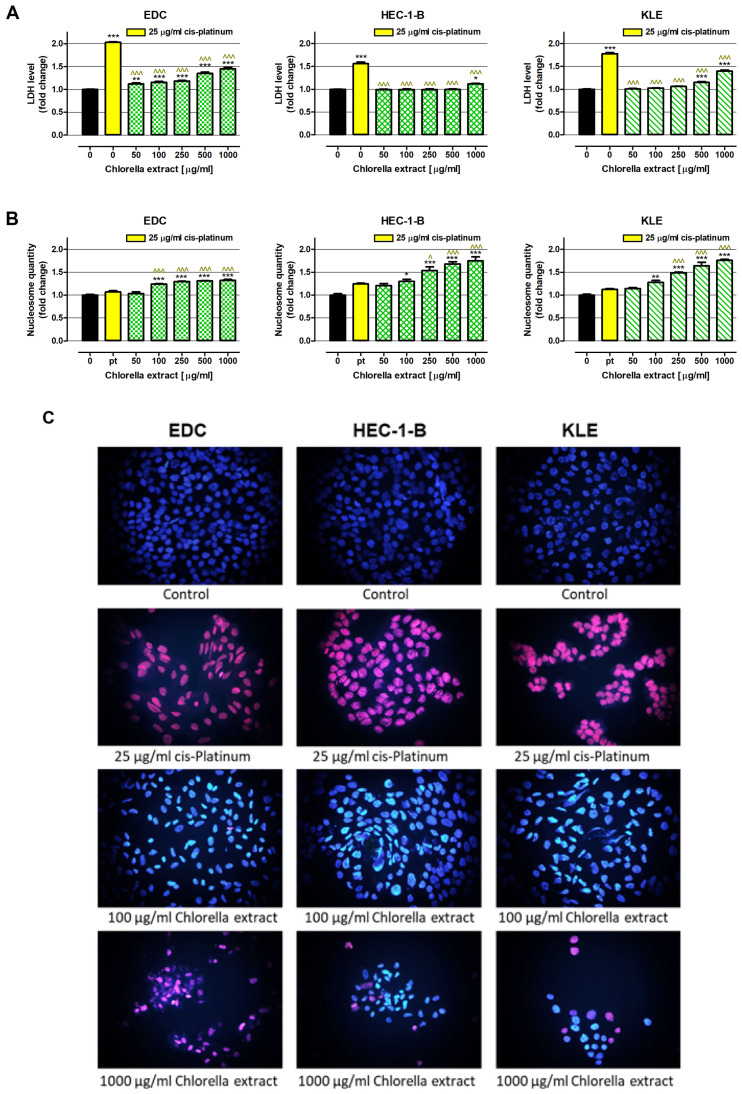
Cell Death Induction in Human Endometrial Adenocarcinoma-derived EDC Cells and Human Endometrial Adenocarcinoma Cell Lines HEC-1-B and KLE in Response to *C. pyrenoidosa* Extract. Cells were exposed for 48 h to a culture medium alone (control), or cis-platinum at a concentration of 25 µg/mL (positive control), or chlorella extract in concentrations of 50, 100, 250, 500 and 1000 μg/mL. Cytotoxic effect of chlorella extract was determined using the LDH assay (**A**), while extract proapoptotic ability was determined by Cell Death Detection ELISA (**B**). Results collected from LDH assay and ELISA are presented as mean ± SEM of at least 3 measurements. Statistically significant differences compared to the control at *p* < 0.05 (*), *p* < 0.01 (**), *p* < 0.001 (***). Statistically significant differences compared to the cis-platinum (positive control) at *p* < 0.05 (^), *p* < 0.001 (^^^). One-way ANOVA test; post-hoc test: Tukey. (**C**) Cell death induction in examined cell lines EDC, HEC-1-B and KLE in response to chlorella extract at concentrations of 100 and 1000 µg/mL or cis-platinum at a concentration of 25 µg/mL was visualized by nuclear double staining. Representative pictures obtained from fluorescence microscopy at magnification 400× are presented. Numerical data are presented in Table S3.

As presented in Figure 3A, chlorella extract destroys cancer cell membranes; however, the effect was different depending on the investigated cell lines. Chlorella extract in the whole range of tested concentrations induced cytotoxicity in EDC cells, which was indicated by elevated release of LDH compared to untreated cells (1.12-fold, 50 µg/mL; 1.16-fold, 100 µg/mL; 1.18-fold, 250 µg/mL; 1.35-fold, 500 µg/mL; 1.45-fold 1000 µg/mL). Similarly, chlorella extract at concentrations of 500 µg/mL and 1000 µg/mL increased LDH levels in culture medium collected from KLE cells compared to the control by 1.15 times and 1.40 times, respectively. In the case of HEC-1-B cells, the cytotoxic influence of the extract was observed only at the highest tested concentration, which increased LDH release by 1.12 times. Used as a positive control, cis-platinum at a concentration of 25 µg/mL revealed significantly stronger cytotoxic effects than the tested extract in all cell lines investigated.

As presented in Figure 3B, chlorella extract at concentrations ranging from 100 µg/mL to 1000 µg/mL induced apoptosis in all investigated cell lines, and the observed effect was dose dependent. The most significant changes were recorded in HEC-1-B and KLE cells, wherein the extract increased the number of nucleosomes (products of DNA degradation and a marker of apoptosis), which changed by 1.30 times and 1.28 times after exposure to 100 µg/mL of the extract and 1.75 times and 1.76 times in response to 1000 µg/mL. In the case of EDC cells, the amounts of nucleosomes increased by 1.24 times and 1.32 times after treatment with the extract at concentrations of 100 and 1000 µg/mL. Cis-platinum at a concentration of 25 µg/mL was not able to induce programed cell death in the investigated cell lines.

Discovered cell death induction in response to chlorella extract was visualized using nuclear double staining. Microscopy evaluation confirmed earlier observations, which showed both necrosis as well as apoptosis induction on tested cell lines treated with chlorella extract (Figure 3C). As previously described, the type of cell death was dependent on cell lines. In the case of EDC treated with the extract at a concentration of 100 µg/mL, most cells underwent programmed cell death, while only single cells were eliminated due to necrosis. On the contrary, EDC incubation with the extract at a concentration of 1000 µg/mL caused a strong cytotoxic effect that masked apoptosis induction. In the case of commercial cell lines HEC-1-B and KLE, chlorella extract at a concentration of 100 µg/mL triggered only apoptosis, while at a 10-times higher concentration induced both types of cell death, but the number of cells undergoing programmed cell death was dominant over the number of necrotic cells. Moreover, microscopy observation revealed a significant decrease in cancer cell numbers in response to the tested extract.

## 3. Discussion

Chemoprevention as a therapeutic strategy, proposed in 1976 by Sporn, refers to the use of natural or synthetic substances to prevent, inhibit, suppress, or reverse the development of premalignant changes in invasive cancers. This strategy also includes the cure of patients who have undergone successful treatment for primary cancer but are still at increased risk of disease recurrence [46]. In contrast to chemotherapy, chemoprevention interferes with the early stages of carcinogenesis, primarily the processes of cancer initiation and promotion.

A key aspect of chemoprevention is the use of substances capable of effectively eliminating cancer cells while having no or negligible toxicity towards normal cells. Our earlier studies demonstrated that water extract of *C. pyrenoidosa* did not impact on viability, proliferation or morphology of human colon epithelial cell line CCD841 CoN [11] and human skin fibroblasts [7]. The lack of side effects of chlorella extract on indicated normal cells was proven in a wide range of tested concentrations and time of treatments. CCD841 CoN cells were exposed to *C. pyrenoidosa* extracts at concentrations from 10 µg/mL to 1000 µg/mL [11], while HSF cells were treated with the extract at concentrations from 2.5 µg/mL to 1000 µg/mL [7]. In both studies, data were collected after 24 and 48 h (LDH assay; cytotoxic examination), 48 h (BrdU assay; impact on DNA synthesis, MGG staining; changes in morphology), and 96 h (MTT assay; impact on cell metabolic activity) [7,11].

In the presented study, using a similar panel of research techniques and the same aqueous extract of *C. pyrenoidosa* at concentrations ranging from 50 to 1000 μg/mL, the second key aspect of a potential chemoprotectant was determined in the context of endo-metrial cancer. Performed studies demonstrated the great anticancer potential of the tested extract in both human endometrial adenocarcinoma-derived EDC cells and human endometrial adenocarcinoma cell lines HEC-1-B and KLE. The beneficial effect of *C. pyrenoidosa* extract was associated with inhibition of cancer cell metabolic activity and DNA synthesis, inhibition of cancer migratory capacity, as well as induction of cancer cell death by apoptosis and/or damage to cell membranes. Moreover, similar to our earlier studies, which revealed the chemopreventive properties of the same chlorella extract against human colon cancer HT-29 cells [11] and human breast cancer T47D cells [7], the observed changes were dose dependent. Unfortunately, as mentioned before, there is a lack of studies showing the influence of chlorella products on the prevention and/or treatment of endometrial cancer. This increases the importance of our research but at the same time prevents us comparing the obtained data with the scientific observations of other researchers.

Nevertheless, it is worth mentioning that there were some differences in the response of the investigated cell lines to *C. pyrenoidosa* extract. HEC-1-B cells were the most sensitive to reduction in metabolic activity, while KLE cells responded the most strongly to inhibition of DNA synthesis. Simultaneously, both KLE and HEC-1-B cells underwent apoptosis triggered by the extract. Moreover, commercial cell lines were also sensitive to the antimigratory influence of chlorella extract; however, the KLE cell line better responded to lower concentrations of the tested compounds. On the other hand, in most experiments, EDC cells were the most resistant to chlorella extract, except for the LDH assay, which revealed significant cytotoxicity in response to the extract over the entire range of tested concentrations. Differences in cell reactions to the investigated compounds seem to be associated with cancer grade. The most significant changes were noted in the KLE cells, histologically classified as a poorly differentiated grade 3 (G3) endometrial tumor. This cell line was derived from endometrial cancer metastasis to the colon. Slightly lower effectiveness of the investigated extract was observed in HEC-1-B cells—histologically consistent with a moderately differentiated grade 2 (G2) endometrial tumor [47,48,49,50]. At the same time, the cells most resistant to chlorella extract were EDC cells derived from the grade 1 endometrial adenocarcinoma. Considering the above, and the earlier-mentioned lack of side effects on normal cells [7,11], it appears that the effectiveness of *C. pyrenoidosa* extract intensifies with increasing cancer advancement and malignancy [47,48,49,50]. This action profile would support the selectivity and therefore the safety of the therapeutic use of the investigated extract.

Analyzing the results collected from well-described commercial human endometrial adenocarcinoma cell lines [47,48,49,50], it is worth paying attention to the pro-apoptotic properties of the investigated extract. While the antiproliferative and antimigratory activity of the *C. pyrenoidosa* extract enhanced with increasing cancer stage, the ability to induce programmed cell death was similar in both cell lines. This is surprising in light of the p53 status in KLE and HEC-1-B cells. KLE cells with wild-type p53 should be more sensitive to apoptosis induction than HEC-1-B with homozygous point mutations in p53 (Arg248Gln) (which significantly alters the biological function of this protein, primarily impacting its ability to bind DNA and regulate gene expression), including those associated with cell viability [51]. The lack of changes in the KLE and HEC-1-B responses to apoptosis induction by chlorella extract suggested that programmed cell death in the indicated cell lines occurs in a way independent of the p53 protein. Of course, this hypothesis requires further verification.

It is worth highlighting that all investigated cell lines readily underwent apoptosis in response to *C. pyrenoidosa* extract. In the case of necrosis, only EDC cells were susceptible to the cytotoxic effects of the extract, while KLE and HEC-1-B cells only entered this cell death pathway after exposure to the higher extract concentrations. Thus, we can conclude that the preferred form of response of the analyzed cells to the tested compounds was apoptosis. The *C pyrenoidosa* extract’s greater predisposition to induce apoptosis than necrosis is a desirable feature in the context of its potential use in oncology, since apoptosis is a programmed, strictly controlled, and ordered cell death that removes damaged or abnormal cells without inducing inflammation, which distinguishes it from necrosis, associated with cell damage and inflammation. Moreover, apoptosis is the most effective and fastest way to eliminate unwanted cells; it proceeds 20 times faster than mitosis. Furthermore, apoptosis allows for the removal of cancer cells without disturbing the neighboring normal cells [52,53]. 

Last but not least is the chemical nature of the *C. pyrenoidosa* extract, which could explain its great anticancer properties against human endometrial cancer cells. The tests performed revealed that the extract consisted mainly of sugars (42.32%), proteins (26.86%) and nucleic acids (24.43%). Despite limited data on the extract composition, due to the method of its acquisition, i.e., water extraction as well as discovered main components, we assume that Chlorella Growth Factor (CGF) is responsible for the described beneficial influence on human endometrial adenocarcinoma cells, since its great anticancer potential was earlier observed in other types of cancers, including breast [7,20,54], colon [11], liver [25,33,55] and lung [19]. CGF is a unique composition of water-soluble substances present in chlorella cells, the most frequently mentioned of which are nucleic acids, free amino acids, peptides, polysaccharides including β-glucans, glycoproteins, vitamins and minerals [56]. Although more than 70 years have passed since the discovery of CGF, there is no standard for its composition, mostly because CCF content is strongly affected by the chlorella species, the culture conditions, and the extraction process [57]. However, the main criterion seems to be the presence of a mixture of proteins, polysaccharides, nucleic acids, and other low-molecular-weight compounds extracted from chlorella cells with water. The investigated extract meets this requirement. However, in-depth chemical analysis is planned for the future, particularly characterizing the polysaccharides and proteins present in the extract, which appear to be crucial to the anticancer abilities of water extract of *C. pyrenoidosa* [7,11,12,14,15,19,20,22,25,33,54,55,56]. Nevertheless, we are convinced that the herein described as well as earlier reported anticancer abilities of the water extract of *C. pyrenoidosa* are the result of the accumulation of different phytochemicals with chemopreventive properties presented in the investigated composition. This conviction is supported by other scientific reports proving that the anticancer effect of different plant extracts has stronger chemopreventive properties than single phytochemicals present in these mixtures. The synergism of these phytochemicals’ biological actions explained why a diet rich in fruits and vegetables revealed stronger health benefits, including chemopreventive effects, than pure anti-cancer substances isolated from these products [58,59,60,61,62].

Although the data presented in this manuscript are basic and many issues require in-depth analysis (including the composition of the extract and its molecular mechanism of action in cells), their value lies in their unique nature. No one has yet assessed the impact of products or substances derived from *Chlorella* species on endometrial cancer (there are no clinical trials or research in in vitro and in vivo models). The obtained results provide a rationale for further studies with a great chance to develop a new chemopreventive strategy for endometrial cancer. However, to complete preclinical studies, it is necessary to (1) characterize all bioactive substances present in the extract; (2) confirm the selectivity of the extract’s action on normal human endometrial cells; (3) understand the molecular mechanisms responsible for the detected beneficial effect of the extract on cancer cells; and (4) check the bioavailability of the extract after oral administration or consider the possibilities of its direct administration to the tumor area.

## 4. Materials and Methods

### 4.1. Reagents

If not indicated otherwise, chemicals used in the study were purchased from Sigma-Aldrich Co. LLC. (St. Louis, MO, USA).

### 4.2. Chlorella Extracts

Dried chlorella (*Chlorella pyrenoidosa*) was purchased from Green Ways International (Prague, Czech Republic). Nutritional values of *C. pyrenoidosa* commercial products used for the preparation of the investigated extract are shown in Table 2. The powdered chlorella in the amount of 5 g was dissolved in 150 mL of sterile water and then extracted for 24 h on a rotator at room temperature. The resulting mixture was centrifuged (4075× *g*, 10 min, 20 °C) and the collected supernatant was filtered through a microbiological filter. The obtained filtrate was subjected to a freeze-drying process. The obtained lyophilizate was stored at −20 °C. A stock solution of chlorella (25 mg/mL) was prepared by dissolving the lyophilizates in phosphate-buffered saline. The obtained extract was stored at −20 °C. Working solutions of the chlorella extract were prepared by dissolving a stock solution in a culture medium.

### 4.3. Evaluation of Sugar, Protein and Nucleic Acid Amounts in Chlorella Extract

Freeze-dried chlorella extract was resuspended in ddH2O at a concentration of 1 mg/mL and subjected to a basic assessment of chemical composition. Total protein content was examined using a Pierce BCA Protein Assay Kit (Thermo Fisher Scientific, Waltham, MA, USA) following the manufacturer’s instructions. Bovine serum albumin was used as a standard. Total sugar amount was estimated using the phenol-sulfuric acid method [63], using glucose as a standard. Nucleic acids were determined using spectrophotometry [64]. All indicated assessments were based on absorbance measurement. The absorbances were recorded using a microplate reader (BioTek, Highland Park, Winooski, VT, USA), at 570 nm wavelength for protein content, at 490 nm wavelength for sugars, and at 260 nm wavelength for nucleic acids. The results were expressed as the percentage of chlorella extract.

### 4.4. Commercial Cell Lines

The human endometrial adenocarcinoma cell lines HEC-1-B and KLE were obtained from the American Type Culture Collection (ATCC, Manassas, VA, USA). HEC-1-B cells were grown in ATCC-formulated Eagle’s Minimum Essential Medium, while KLE cells were grown in ATCC-formulated Dulbecco’s Modified Eagle’s Medium/Nutrient Mixture F-12 Ham. Both media were supplemented with penicillin (100 U/mL) and streptomycin (100 μg/mL) and 10% FBS. Cells were maintained in a humidified atmosphere of 95% air and 5% CO_2_ at 37 °C.

### 4.5. Non-Commercial Cell Line

Human endometrial adenocarcinoma-derived EDC cells came from the uterine body of a 61-year-old woman diagnosed with G1 endometrial adenocarcinoma with features of squamous differentiation. Immediately after collection, the tumor fragment was placed in chilled PBS with an antibiotic antimycotic solution and transported to the in vitro laboratory. In a laminar flow cabinet, blood clots and vessels were removed from the tumor. Next, the tumor was cut with a scalpel into small fragments and washed again with PBS with an antibiotic antimycotic solution. Then, tumor fragments were suspended in DMEM medium supplemented with collagenase 500 µg/mL and DNase 25 U/mL and incubated for 45 min at RT with constant mixing. Digestion mixture was removed by centrifugation (250× *g* for 10 min). The cell pellet was suspended in Dulbecco’s Modified Eagle’s Medium supplemented with penicillin (100 U/mL) and streptomycin (100 μg/mL) and 10% FBS. Cells were maintained in a humidified atmosphere of 95% air and 5% CO_2_ at 37 °C. The culture was maintained until passage 68 without a significant decrease in proliferative potential. Cells at earlier passages were systematically banked in liquid nitrogen. For experiments, EDC cells from passages 20 to 25 were used.

The experimental protocol was approved on 23 February 2023, by the Bioethics Committee at the Lublin Medical University, Lublin, Poland (Resolution No KE-0254/35/02/2023).

### 4.6. Evaluation of Chemopreventive Abilities of Chlorella Extract

Cells were seeded on 96-well microplates (MTT, LDH, BrdU assays and ELISA) or petri dishes (wound assay, nuclear double staining) at a density of 1 × 105 cells/mL (for ELISA, LDH, nuclear double staining and wound assays), at a density of 5 × 104 cells/mL (for BrdU assay) and 3 × 104 cells/mL (for MTT assay). Cells were exposed to chlorella extract in the following concentrations: 50, 100, 250, 500 and 1000 μg/mL. After certain exposure times, the extract influence on cell viability, proliferation and migratory capacity was examined using the assays indicated below.

#### 4.6.1. Examination of Cell Metabolic Activity—MTT Assay

After 96 h of cell treatment with chlorella extract, an MTT assay was conducted. In the first step, the MTT solution (5 mg/mL in PBS) was added to the cells for 3 h. Resultant crystals were solubilized overnight in SDS buffer, pH 7.4 (10% SDS in 0.01 N HCl), and the product was quantified spectrophotometrically by measuring the absorbance at 570 nm wavelength on the BioTek ELx800 microplate reader. The results were presented as a percentage of the metabolic activity of cells treated with chlorella extract versus cells grown in the control medium (indicated as 100%).

#### 4.6.2. Evaluation of Cell Proliferation—BrdU Assay

After 48 h of cell treatment with chlorella extract, cell proliferation was assessed by the colorimetric immunoassay, Cell Proliferation ELISA BrdU, according to the manufacturer’s instructions (Roche Diagnostics GmbH, Penzberg, Germany). Absorbance was measured at 450 nm wavelength on the BioTek ELx800 microplate reader. The results were presented as a percentage of the BrdU incorporation of DNA in cells treated with chlorella extract versus cells grown in the control medium (indicated as 100%).

#### 4.6.3. Assessment of Cell Membrane Integrity—LDH Assay

After 48 h of cell treatment with chlorella extract or cis-platinum (25 µg/mL) the culture medium was transferred to new 96-well microplates, which were used to perform the LDH assay following the manufacturer’s instructions (In Vitro Toxicology Assay Kit Lactate Dehydrogenase Based, Sigma). Absorbance was examined at 450 nm wavelength on the BioTek ELx800 microplate reader. The results were presented as the fold change of LDH released from cells treated with chlorella extract versus cells grown in the control medium (indicated as 1).

#### 4.6.4. Examination of Apoptosis—ELISA

After 48 h of cell treatment with chlorella extract or cis-platinum (25 µg/mL), apoptosis induction was assessed using a Cell Death Detection ELISA PLUS kit (Roche Diagnostics, Mannheim, Germany) according to the manufacturer’s instructions. Absorbance was measured at 405 nm wavelength on the BioTek ELx800 microplate reader. The results were presented as the fold change of nucleosomes in cells treated with chlorella extract versus cells grown in the control medium (indicated as 1).

#### 4.6.5. Visualization of Cell Death—Nuclear Double Staining

After 48 h of cell treatment with chlorella extract at concentrations of 100 and 1000 µg/mL or cis-platinum at a concentration of 25 µg/mL, cell death was visualized by nuclear double staining with a fluorochrome mixture: Hoechst 33342 (0.24 mg/mL) and propidium iodide (0.15 mg/mL). After 5 min of cell incubation with the indicated fluorochromes, cells were examined under a fluorescence microscope (Olympus BX51 System Microscope, Olympus Optical Co., Ltd., Tokyo, Japan). Cell images were captured using CellFamily AnalySIS software version 3.3 (Matrix Optics, Subang Jaya, Malaysia).

#### 4.6.6. Examination of Cell Migratory Capacity—Wound Assay

The day after the cells were seeded on Petri dishes the culture medium was removed and the cell monolayer was scratched by a pipette tip to create a wound (cell-free area to monitor cell migration). To remove the detached cells, the dish was rinsed with PBS. After that, cells were exposed to chlorella extract at the following concentrations: 250, 500 and 1000 μg/mL. To assess cell migration capacity, after 48 h of treatment the dishes were stained according to the May–Grünwald–Giemsa (MGG) method. In the first step, the culture medium was replaced by the May–Grünwald solution. After 3 min of incubation, the dye solution was diluted in water twice and incubation was continued for the next 3 min. Then, the May–Grünwald solution was replaced by Giemsa diluted in water 20 times, and stained for the next 30 min. After removing the excess dye and drying preparations, cell migratory capacity was observed in a light microscope MW 50 (OPTA-TECH, Warsaw, Poland), while micrographs were prepared using Capture software version 2.2.1 (OPTA-TECH, Warsaw, Poland). The cell migration to the wound was determined by examination of the wound area overgrown with cells using ImageJ software extended with an MRI Wound Healing Tool.

### 4.7. Statistical Analysis

The data were presented as the mean ± SEM. One way-ANOVA with the Dunnett or Tukey post-hoc tests and column statistics were used for comparisons. Significance was accepted at *p* < 0.05. The IC50 value (concentration leading to 50% inhibition of cell viability/proliferation compared to the control) was calculated using GraphPad PRISM v. 5.0.

## 5. Conclusions

The results obtained revealed for the first time the chemopreventive properties of the water extract of *C. pyrenoidosa* in an in vitro model of endometrial cancer. We discovered beneficial effects of the chlorella extract based on inhibition of cancer cell viability, proliferation and migratory capacity, as well as induction of both cytotoxicity and programmed cell death. The range of chemoprevention seemed to intensify with increasing cancer stage and malignancy. This observation, together with a previously proven lack of any side effects in human skin fibroblasts and human colon epithelial cell lines, confirms the great chemopreventive abilities of the investigated extract and provides hope for its further therapeutic use. The collected data encourage future research, including clinical as well as detailed chemical evaluation to specify the phytochemical composition of the tested extracts.

## Figures and Tables

**Table 1:** Basic chemical composition of chlorella water extract. The percentage total amounts of sugars, proteins and nucleic acids were determined using methods based on the measurement of absorbance at wavelengths of 570 nm, 490 nm and 260 nm, respectively.

	Total Proteins (Mean ± SD)	Total Sugar (Mean ± SD)	Total Nucleic Acids (Mean ± SD)
Amount in water extract of *Chlorella pyrenoidosa*	26.86 ± 3.49%	42.32 ± 2.01%	24.43 ± 0.99%

**Table 2:** Nutritional values of the *Chlorella pyrenoidosa* commercial product used for the preparation of the examined extract. Nutritional value was shown as per 100 g dry weight of the product. The presented data were prepared based on the manufacturer‘s information.

	Commercial Product of *Chlorella pyrenoidosa*
Energy	1634 kJ, 389 kcal
Proteins	54.13 ± 0.85 g
Fat	11.60 ± 0.41 g
Carbohydrates	11.63 ± 1.55 g
Total Dietary Fiber	10.84 ± 0.73 g

## Data Availability

All data analyzed during this study are included in this article. Further inquiries can be directed to the corresponding author.

## Supplementary Materials

The following supporting information can be downloaded at https://www.mdpi.com/article/10.3390/ijms26189142/s1.
