# Supplementary material for: First Look at Chemopreventive Properties of Chlorella pyrenoidosa Water Extract in Human Endometrial Adenocarcinoma Cells—Preliminary In Vitro Study
Source: Int J Mol Sci. 2025 Sep 19;26(18):9142. doi: 10.3390/ijms26189142 (PMC12471056; doi:10.3390/ijms26189142)
Supplement: Supplementary file 1 [file ijms-26-09142-s001.zip › ijms-3828576-supplementary.pdf]

Table S1. Antiproliferative effect of *C. pyrenoidosa* extract in human endometrial cancer cells (EDC, HEC-1-B, KLE). The cell metabolic activity was determined using the MTT assay, while DNA synthesis was assessed using the BrdU test. Results are presented as mean of % of control ± SD of at least 4 measurements.

**Extract concentrations**	**EDC**		**HEC-1-B**		**KLE**	
	**MTT assay**	**BrdU assay**	**MTT assay**	**BrdU assay**	**MTT assay**	**BrdU assay**
**0 μg/ml**	100.0 ± 0.9%	100.0 ± 1.7%	100.0 ± 1.3%	100.0 ± 2.7%	100.0 ± 3.3%	100.0 ± 1.5%
**50 μg/ml**	71.4 ± 2.5%	83.4 ± 1.5%	56.8 ± 3.0%	94.0 ± 0.9%	73.6 ± 2.1%	91.6 ± 1.6%
**100 μg/ml**	60.9 ± 1.4%	75.5 ± 1.5%	54.5 ± 0.8%	90.7 ± 2.9%	64.5 ± 1.6%	82.0 ± 3.0%
**250 μg/ml**	53.8 ± 1.9%	70.6 ± 0.6%	37.7 ± 2.1%	87.1 ± 2.0%	40.0 ± 4.4%	59.3 ± 4.4%
**500 μg/ml**	47.2 ± 0.9%	64.6 ± 1.0%	12.9 ± 0.8%	66.7 ± 1.3%	17.9 ± 2.2%	36.0 ± 3.0%
**1000 μg/ml**	43.0 ± 1.5%	58.6 ± 2.2%	0.4 ± 0.1%	20.9 ± 1.6%	1.0 ± 0.4%	7.7 ± 3.7%

Table S2. Antimigratory effect of *C. pyrenoidosa* extract in human endometrial cancer cells (EDC, HEC-1-B, KLE). The migratory capacity of cancer cells was determined by examination of the wound area overgrown with cells in response to the chlorella extract using Image J software. Results are presented as mean of % of control ± SD of 4 measurements.

**Extract concentrations**	**EDC**	**HEC-1-B**	**KLE**
**0 μg/ml**	100.0 ± 2.0%	100.0 ± 5.4%	100.0 ± 4.6%
**250 μg/ml**	96.1 ± 3.0%	86.7 ± 3.1%	83.2 ± 11.6%
**500 μg/ml**	90.8 ± 5.5%	81.9 ± 8.0%	69.7 ± 8.3%
**1000 μg/ml**	79.2 ± 1.9%	60.0 ± 5.8%	56.4 ± 8.0%

Table S3. Cell death induction in human endometrial cancer cells (EDC, HEC-1-B, KLE) in response to *C. pyrenoidosa* extract. Cytotoxic effect of chlorella extract was determined using the LDH assay while extract proapoptotic ability was determined by Cell Death Detection ELISA. As a positive control, was used cis-platinum at a concentration of 25 μg/ml. Results are presented as mean of % of control ± SD of at least 3 measurements.

**Extract concentrations**	**EDC**		**HEC-1-B**		**KLE**	
	**LDH assay**	**ELISA**	**LDH assay**	**ELISA**	**LDH assay**	**ELISA**
**0 μg/ml**	100.0 ± 1.5%	100.0 ± 2.7%	100.0 ± 1.1%	100.0 ± 5.1%	100.0 ± 1.3%	100.0 ± 3.6%
**50 μg/ml**	111.9 ± 4.8%	103.3 ± 7.3%	99.2 ± 2.4%	120.8 ± 7.4%	101.0 ± 1.6%	114.3 ± 4.0%
**100 μg/ml**	115.8 ± 3.9%	124.1 ± 2.5%	99.5 ± 2.2%	129.8 ± 8.2%	102.8 ± 0.6%	127.6 ± 7.5%
**250 μg/ml**	118.4 ± 2.4%	129.5 ± 1.9%	99.5 ± 3.0%	153.8 ± 14.3%	106.4 ± 1.6%	148.8 ± 2.9%
**500 μg/ml**	135.2 ± 5.6%	131.1 ± 1.3%	99.7 ± 1.9%	168.3 ± 8.3%	115.1 ± 3.4%	164.4 ± 13.1%
**1000 μg/ml**	145.1 ± 6.1%	132.4 ± 3.7%	111.8 ± 2.6%	175.0 ± 15.3%	139.9 ± 3.8%	176.4 ± 3.7%
**25 μg/ml cis-platinum**	203.1 ± 2.6%	107.0 ± 4.7%	156.1 ± 7.2%	124.2 ± 3.8%	178.0 ± 5.0%	112.4 ± 3.8%
